# Physical activity and heart rate monitoring in Fontan patients – Should we recommend activities in higher intensities?

**DOI:** 10.1371/journal.pone.0228255

**Published:** 2020-01-30

**Authors:** Julian Alexander Härtel, Ulrike Herberg, Thomas Jung, Christian Winkler, Johannes Breuer, Nicole Müller

**Affiliations:** Department for Paediatric Cardiology, University Hospital Bonn, Bonn, Germany; University of Maiduguri College of Medical Sciences, NIGERIA

## Abstract

**Objective:**

Exercise capacity is impaired in Fontan palliated patients. The change in daily activity behaviour with an increase in sedentary lifestyle results in low physical activity levels. This might have a greater impact on patients with chronic heart disease in contrast to healthy controls. For a better understanding, we compared data from cardiopulmonary exercise testing (CPET) with heart rates and physical activity in daily life.

**Methods:**

21 Fontan patients and 20 age, sex and BMI matched controls underwent CPET and 5 days of daily life activity tracking with a triaxial accelerometer (wGT3x-BT, Actigraph) including heart rate monitoring with an optical heart rate sensor.

**Results:**

27% of our Fontan teenagers and 71% of the Fontan adults reached the specific WHO recommendations for moderate to vigorous physical activity (MVPA) during everyday life (EDL), without differences to controls. There was a strong correlation between MVPA and $\dot{V}O_{2\mathrm{peak}}$ for all Fontan patients (p = 0.0035, Pearson r = 0.788). Daily MVPA correlated to peak oxygen uptake and lactate production. Up to workloads of 2 W/kg and in daily life heart rates in Fontan patients were similar to controls.

**Conclusions:**

Daily MVPA is alarmingly low without any differences between Fontan patients and controls. Heart rate behaviour was similar and does not seem to be a limiting factor for physical activity in daily life. Higher intensity activities should be implemented regularly in EDL for Fontan patients. Proof is needed as to whether sports in moderate or possibly even in vigorous activity (e.g. high-intensity interval training) improve exercise capacity the most.

## Introduction

In Germany, 200 children are born annually with a complex heart defect leading to a univentricular circulation [1]. Staged palliative surgery resulting in Fontan circulation, enables life expectancy until adulthood. While the short- and median-term survival in Fontan palliated children has improved considerably during the last decades, cardiac function and exercise capacity are still reduced compared to healthy children and adults. Previous studies, investigating exercise capacity showed a relevant impairment with approximately 60% of $\dot{V}O_{2\mathrm{peak}}$ in patients after Fontan palliation compared to age-matched healthy subjects [2–4]. Besides cardiac function, including chronotropic incompetence, other causes of this impairment (such as lung function, skeletal muscle, and endothelial dysfunction) remain undefined [5–8].

There appears to be a psychosocial component, with parental anxiety often leading to overprotection of children with complex heart defects, probably resulting in low physical activity levels [9, 10]. This is problematic, since an inactive, sedentary lifestyle is becoming more common in childhood and can lead to further restrictions in cardiac function and physical capacity especially in Fontan patients, since this patient group is assumed to have a disproportionately loss of cardiopulmonary capacity at younger age [9].

Daily physical activity could be one of the most important factors for improving physical capacity and quality of life.

Unfortunately, due to lack of data, there are no appropriate guideline recommendations for physical activity and sport in children with univentricular hearts. Sport is recommended, but details about type, intensity and duration remain unclear [11]. This frequently leads to exclusion of children from school and team sports, and subsequently, lower activity levels in everyday life (EDL), even though it has been shown that physical exercise is safe for Fontan patients [9, 12, 13].

The aim of this study is to evaluate daily physical activity with associated heart rates, measured by a triaxial accelerometer and an optical heart rate monitor, and its correlation with physical capacity (oxygen uptake, lactate i.a.) in Fontan patients. To our knowledge, it is the first study investigating heart rate behaviour in Fontan patients during everyday life.

## Methods

### Participants

We investigated 21 Fontan patients and 20 age-, sex- and BMI-matched controls (Table 1).

**Table 1:** Participants characteristics.

		Fontan (*n = 21*)	Control (*n = 20*)
Age [years]		18 [14,31]	18 [14,33]
Gender [f / m]		9 / 12	9 / 11
Body mass index, BMI [kg/m^2^]		22.9 ± 3.77*	22.26 ± 3.32*
Body surface area, Mosteller [m^2^]		1.71 ± 0.23*	1.77 ± 0.23*
Peripheral oxygen saturation, SpO_2_ [%]		98.85 ± 1,09*	92.4 ± 4.06*
Age at Fontan surgery [months]		41 .9 ± 28.44*	
Years since Fontan surgery [years]		15.76 ± 3.0*	
Type of Fontan circulation	fenestration	2	
Underlying cardiac defect			
	I. Functional left ventricle		
	Tricuspid atresia	3	
	Double Inlet Left Ventricle (DILV)	4	
	Single Inlet Left Ventricle	1	
	II. Functional right ventricle		
	Hypoplastic Left Heart Syndrome	7	
	Double Outlet Right Ventricle (DORV)	2	
	Single ventricle with ccTGA	4	
Pacemakers	2 x AAIR, 1 x VVIR	3	
Drugs			
	I. Cardioselective β-blocker	4	
	II. ACE-inhibitors	8	
	III. Both	2	

*mean ± SD

Inclusion criteria: (1) age ≥14 years at the time of investigation, (2) New York Heart Association (NYHA) class I or II, (3) mental and physical ability for cardiopulmonary exercise test (CPET) on a bicycle ergometer.

Exclusion criteria: (1) failing Fontan, (2) mental disability, (3) $\dot{V}O_{2\mathrm{peak}}$ <45% of predicted values, (4) pregnancy.

Additional exclusion criteria for controls: (1) cardiovascular disorders, (2) smoking, (3) athletes.

This study was approved by the local ethics committee (application number 335/14) and corresponded with the Declaration of Helsinki.

All participants/parents gave written informed consent.

### Study procedure

41 participants underwent CPET until subjective exhaustion, while blood pressure, BORG scale and capillary blood probes from the earlobe were obtained every two minutes.

After CPET, all participants received an activity tracker and a heart rate monitor to record at least five days of everyday activity.

### Exercise testing

CPET was executed on a computer-controlled sitting bicycle ergometer (ERG 911 Plus, Ergosana GmbH Schiller, Bitz/Germany) with a breath-by-breath gas exchange analysis (Metamax 3B with MetaSoft Studio Software v. 5.4 for analysis, Cortex Biophysik GmbH, Leipzig/Germany). $\dot{V}O_{2\mathrm{peak}}$ was defined as the highest mean uptake of any 30-second interval during the exercise.

**Fig 1:**
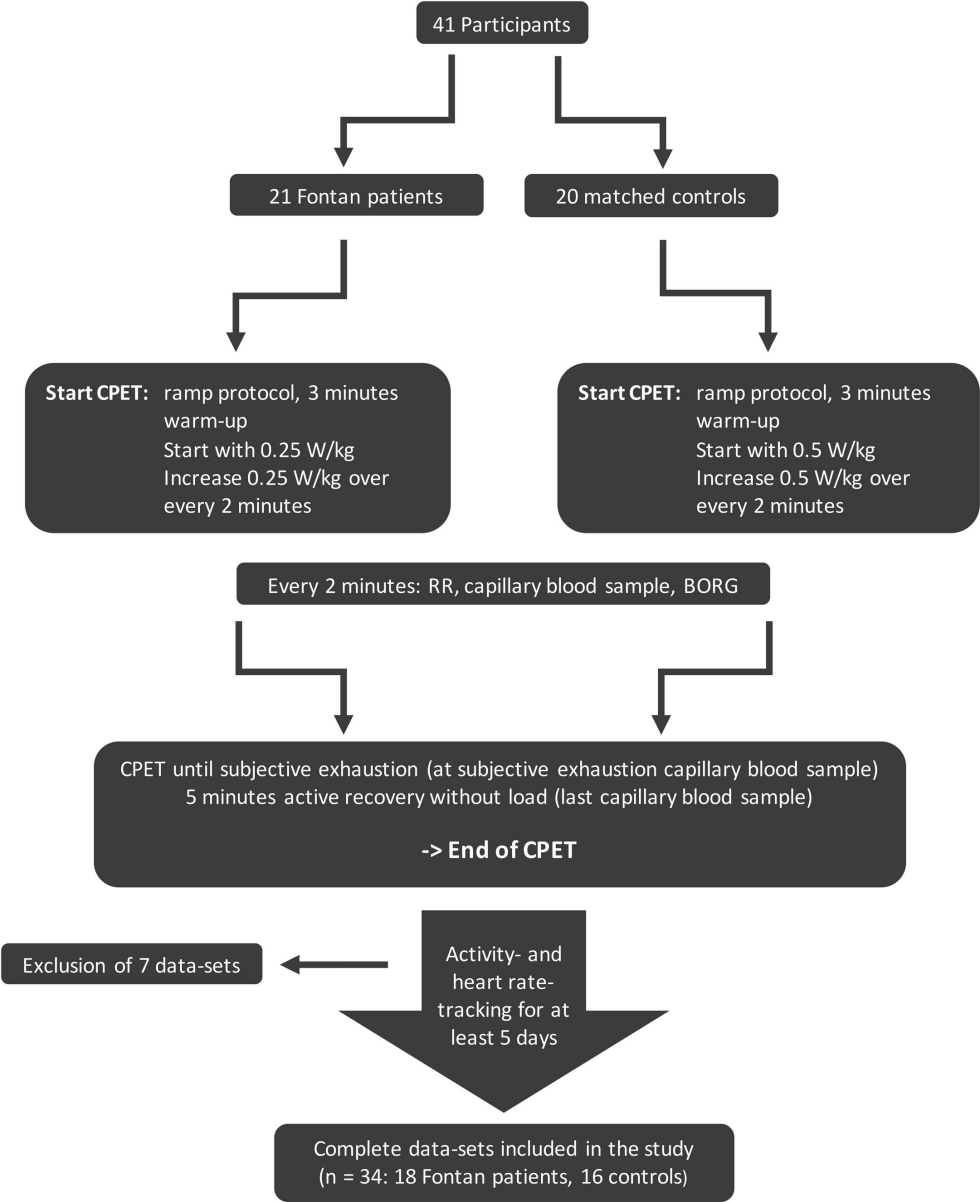
Study procedure and completed tests.

A ramp protocol with increasing workload was used until subjective exhaustion was reached according to the BORG scale and when cadence ≥ 60 rpm could not be sustained. It was followed by a 5-minute recovery period (Fig 1).

A continuous 12-lead ECG was performed (Custo Diagnostics, V. 5.4, Custo med GmbH, Ottobrunn/Germany) and blood pressure was measured every two minutes (Tango M2, SunTech Medical Inc., Morrisville/USA). Transcutaneous oxygen saturation was constantly measured by a pulse oximeter (V100, GE Healthcare, Chicago/USA). Capillary blood probes were measured by LabTrend (BST Bio Sensor Technology GmbH, Berlin/Germany).

### Daily activity and continuous heart rate monitoring

All participants received the triaxial accelerometer, Actigraph wGT3x-BT (Actigraph LLC., Pensacola/USA), validated by Lee et al. or Chu et al. (i.a.) [14–16] and an optical heart rate monitor, OH1 (Polar Electro, Kempele/Finland). The heart rate monitor was connected via Bluetooth to the Actigraph wGT3x-BT, which stored the data. For at least 5 days, including one weekend day, study subjects wore the accelerometer on their right hip using an elastic belt, and the OH1 on the inside of the right upper arm.

Activity data were included when a minimum wear-time of 330 minutes per day over five days was reached (sampling rate 30 Hz).

Data analysis was performed with the ActiLife 6 software (V. 6.13.3, Actigraph LLC., Pensacola/USA). Accelerometer-based assessments of physical activity (counts per minute) with an epoch length of 10 seconds were converted in different activity intensity categories, using cut-off points by Freedson et al. [17].

Moderate or higher intensity activities are classified as moderate-to-vigorous physical activity (MVPA). Daily activity is defined as the mean value of the activity data on the specific days, given as percent of the wear time.

Due to age-specific activity recommendations, groups were subdivided into teenagers (14–18 years) and adults (>18 years) [18].

### Statistical analysis

All analyses were done with GraphPad Prism (V. 7.0e, GraphPad Software, La Jolla/USA), using the paired student’s t-test for exercise data. After testing for normal distribution using the Shapiro-Wilk normality test, the student’s unpaired t-test was used to compare daily physical activity and heart rate.

Linear regression was used to detect correlations between parameters, and Pearson’s correlation coefficient was used to measure the correlation strength.

Quantitative values are given in median (min, max) unless otherwise described.

P-values ≤0.05 were classified as significant.

## Results

All 41 participants completed the cardiopulmonary exercise test.

Seven activity- and heart rate data sets were excluded (three Fontan and four controls) due to insufficient wear-time or technical problems. 34 complete data sets including results of CPET, activity tracking and heart rate monitoring, from 18 Fontan patients and 16 healthy controls were evaluated (Fig 1).

### Exercise capacity (*n = 41*)

The Fontan group achieved 63% of the exercise capacity, expressed as median $\dot{V}O_{2\mathrm{peak}}$, compared to controls (25 (17, 32) ml/min/kg vs. 40 (25, 56) ml/min/kg, p ≤0.0001). The peak workload, expressed as median value of power per weight (W/kg), was 1.8 (1.3, 2.3) W/kg in Fontan patients compared to 3.3 (1.8, 4.2) W/kg in controls (55% of the peak workload of controls; p ≤0.0001).

Peak capillary lactate levels were significantly lower in Fontan patients 3.4 (2.1, 6.4) mmol/l than in healthy controls 7.35 (3.7, 11.8) mmol/l (p = 0.0008). There was no significant difference in capillary lactate concentrations at rest.

In the following, we compare parameters at similar workloads (0.5 W/kg, 1.0 W/kg, 1.5 W/kg), since peak values only represent individual capacity and have limited comparability.

### Daily activity (*n = 34*)

Detailed activity data is shown in Table 2.

**Table 2:** Activity data.

**Teenagers (<18 years)**				
		*Fontan (n = 11)*	*Control (n = 6)*	*Significance level (p-value)*
∅ daily wear-time	[min]	581 [491, 884]	610 [573, 762]	ns (0.70)
∅ daily steps	[counts/d]	7,033 [2,330, 16,143]	6,204 [5,19, 12,321]	ns (0.88)
∅ time sedentary	[%]	73.6 [60.6, 85.1]	71.8 [65.9, 76.4]	ns (0.96)
∅ time light activity	[%]	20.1 [12.1, 30.3]	20.9 [17.0, 25.7]	ns (0.95)
∅ time MVPA	[%]	6.3 [2.7, 11.3]	6.6 [3.6, 11.7]	ns (0.99)
	[min/d]	42 [16, 97]	39 [23, 83]	ns (0.86)
Subjects achieving WHO recommendations for children [18]		3/11	2/6	
**Adults (≥18 years)**				
		*Fontan (n = 7)*	*Control (n = 10)*	*Significance level (p-value)*
∅ daily wear-time	[min]	640 [539, 757]	635 [443, 800]	ns (0.54)
∅ daily steps	[counts/d]	7,600 [4,493, 9,678]	6,117 [3,204, 14,657]	ns (0.78)
∅ time sedentary	[%]	68.2 [63.5, 80.6]	71.0 [65.1, 86.9]	ns (0.46)
∅ time light activity	[%]	21.9 [17.2, 29.4]	19.8 [11.1, 26.9]	ns (0.14)
∅ time MVPA	[%]	6.5 [2.2, 11.3]	7.6 [2.0, 14.7]	ns (0.56)
	[min/5d]	217 [75, 351]	181 [75, 509]	ns (0.63)
Subjects achieving WHO recommendations for adults [18]		5/7	8/10	

median and range, ns = not significant

### Teenagers

Fontan patients and controls showed similar levels of physical activity frequencies, intensity, and step counts per day.

The World Health Organization (WHO) recommends a daily minimum of 60 minutes of moderate-to-vigorous physical activity for teenagers [18], which was reached by 27% of Fontan patients compared to 33% of controls.

### Adults

No significant differences in activity frequency or intensity were found between Fontan and control adults.

For adults, the WHO recommends at least 150 minutes of aerobic activity in moderate intensity weekly [18]. Over a five-day tracking period, 71% of Fontan patients reached the recommended MVPA time compared to 80% of controls, with no significant difference between the groups.

### Daily physical activity and exercise capacity

There was a strong, statistically significant correlation between MVPA and $\dot{V}O_{2\mathrm{peak}}$ for all Fontan patients (p = 0.0035, Pearson r = 0.788), but only a positive trend was seen between both parameters for control teenagers (p = 0.053, Pearson r = 0.65).

Peak capillary lactate concentrations did not correlate with MVPA. However, control teenagers with higher daily activities demonstrated a trend for higher peak capillary lactate concentrations and showed the highest $\dot{V}O_{2\mathrm{peak}}$ values.

**Fig 2:**
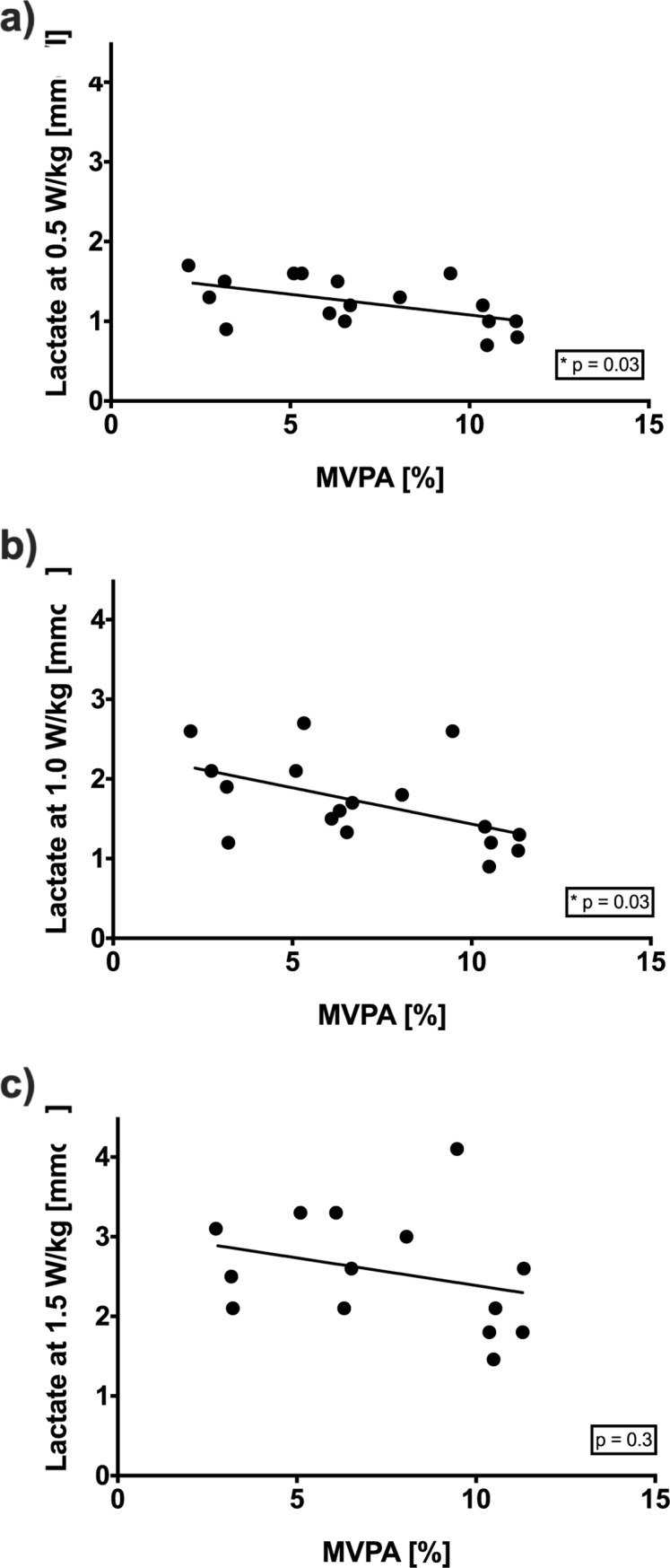
Correlation between capillary lactate levels in Fontan patients at workloads of a) 0.5 W/kg, b) 1.0 W/kg, c) 1.5 W/kg and total measured MVPA in percent.

**Fig 3:**
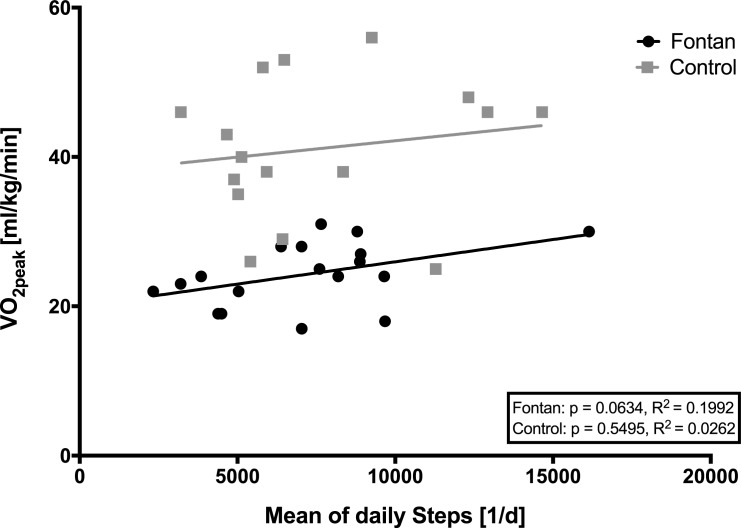
Correlation between $\dot{V}O_{2\mathrm{peak}}$ and mean of daily steps. No statistically significant correlation could be detected.

There was a significant correlation between time spent in MVPA and lactate concentrations in Fontan patients at 0.5 W/kg and 1.0 W/kg, but not at 1.5 W/kg or in controls. Nevertheless, in Fontan patients at 1.5 W/kg, there was a negative relationship trend (Fig 2). Lower intensity activities and number of steps (daily and total) did not significantly correlate with peak exercise parameters in any of the groups (Fig 3).

### Heart rates during CPET and in everyday life

#### CPET

Heart rate data is shown in Table 3.

**Table 3:** Heart rate behaviour during CPET and everyday life (EDL).

a)		*Fontan teenagers*	*Control teenagers*	*Significance level (p-value)*	*Fontan adults*	*Control adults*	*Significance level (p-value)*
CPET Rest	(bpm)	81 [69,95]	84 [72,101]	ns*	73 [56,92]	75 [72,96]	ns*
CPET peak	(bpm)	164 [101,187]	188 [182,204]	0.0178	169 [93,196]	193 [153,206]	0.0185
b)							
∅ EDL	(bpm)	89 ± 17	89 ± 20	ns*	83 ± 16	83 ± 18	ns*
EDL peak	(bpm)	156 [115,194]	179 [148,196]	0.0352	137 [124,168]	169 [132,189]	0.0291
c)							
Proportion EDL peak : CPET peak	%	101 [86,139] (ns) *	97 [81,104] (ns) *		99 [70,133] (ns) *	89 [71,93] (p = 0.0006)	

a) Comparison of heart rates during CPET in the group of teenagers and adults. Heart rates at rest and peak heart rates are compared.

b) Comparison of heart rates in everyday life. Heart rates during EDL are presented as means ± SD

c) Proportion of individual HR_peak_ during EDL and HR_peak_ during CPET in percent. Only adult controls showed significantly higher peak HR during CPET.

*ns = not significant

Resting heart rate (HR_rest_), measured in a reclining position before exercise, did not differ between groups.

**Fig 4:**
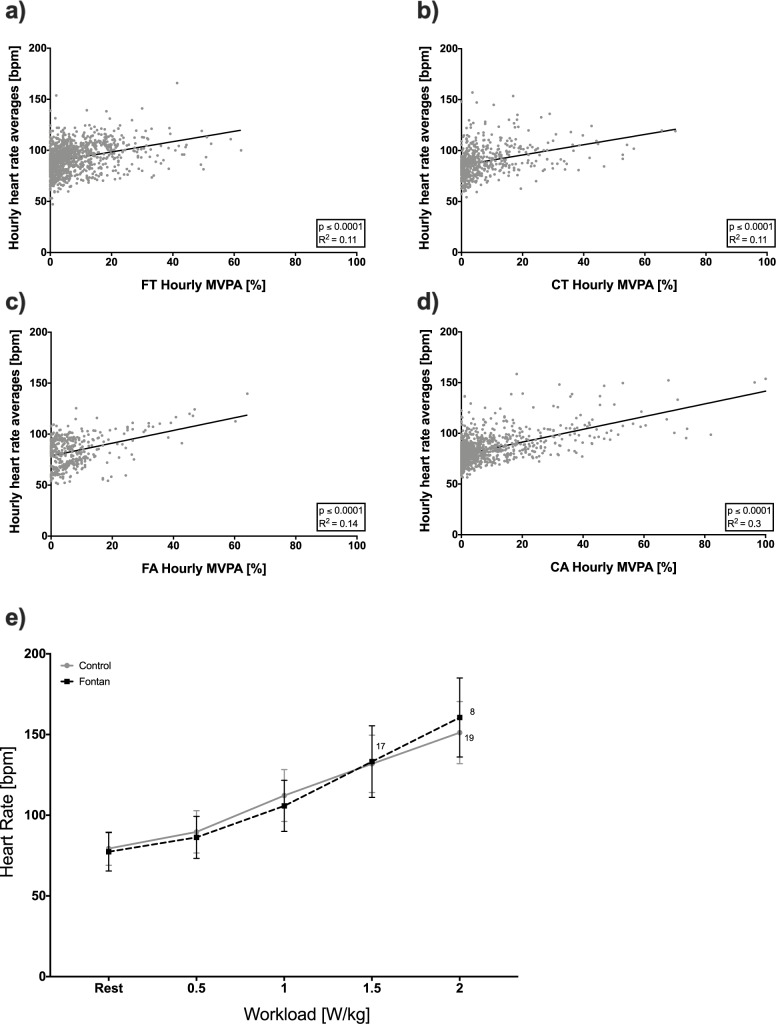
Correlation of hourly MVPA in percent and hourly averages of heart rates. a) Fontan teenagers (FT), b) Control teenagers (CT), c) Fontan adults (FA), d) Control adults (CA), e) Heart rates during CPET. Numbers represent participants reaching the defined workloads.

Heart rates at maximal exhaustion (HR_peak_) differed significantly in teenagers and adults. Teenage and adult Fontan patients only reached 87.2% and 87.6%, respectively, of the peak heart rates of their matched controls. When comparing heart rates at similar workloads, there was no difference between Fontan patients and controls (Fig 4E). Three patients with a pacemaker had no stimulation above 80 bpm, since sinus rhythm showed an appropriate function in higher frequencies.

#### Everyday life

**Fig 5:**
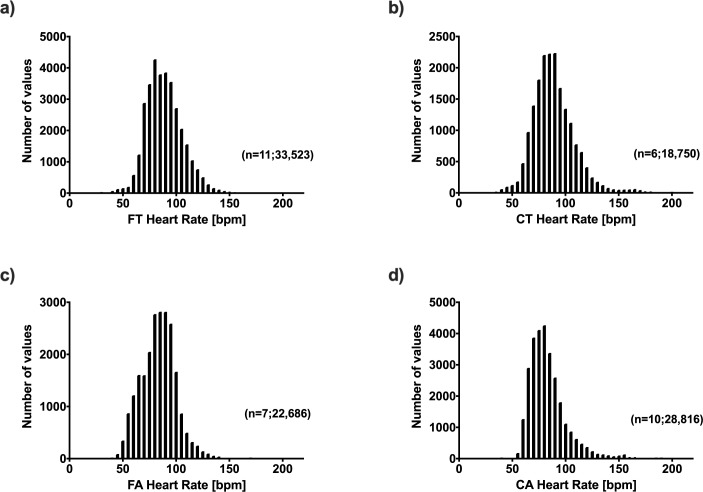
Distribution of heart rate data during everyday life (EDL). With a mean of 89 bpm a) Fontan teenagers (FT) and b) Control teenagers (CT) show similar distributions of heart rates. With a mean of 83 bpm c) Fontan adults (FA) and d) Control adults (CA) also showed similar distributions of heart rates.

Nearly 3,000 minutes of heart rate data could be investigated in every subject. Fig 5 and Table 3 show the distribution of heart rates over the total tracking time.

Average heart rates in the adult groups were significantly different to the teenager groups (p ≤0.0001).

A highly significant correlation between hourly average heart rates and hourly MVPA was seen in 14/18 Fontan patients and 15/16 controls (Fig 4A–4D). The effect sizes did not differ between groups, so that an increase in MVPA resulted in similar increases in average heart rates.

**Fig 6:**
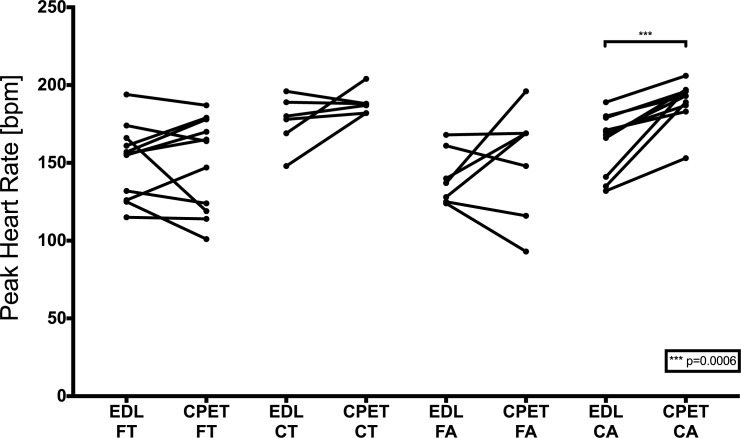
Comparison of peak heart rates in everyday life (EDL) and during CPET. FT = Fontan teenagers, CT = Control teenagers, FA = Fontan adults, CA = Control adults.

Fig 6 illustrates peak heart rates at CPET and in daily life, but significantly higher peak heart rates during CPET compared to peak heart rates during tracking were only found in control adults (Table 3C). In contrast, only three adult Fontan patients demonstrated similar heart rate behaviour, whereas 4/7 had lower or similar peak heart rates during CPET. Both teenager groups reached similar or even higher peak heart rates during everyday life compared to CPET.

## Discussion

To our knowledge, this is the first study comparing activity behaviour and daily heart rates of Fontan patients to matched controls. Although no significant differences were observed between the groups for these parameters, daily activity significantly correlated with exercise parameters such as $\dot{V}O_{2\mathrm{peak}}$ or capillary lactate in Fontan patients.

### Physical activity in everyday life

No significant difference was seen in time spent in MVPA or average step counts per day within the Fontan patients (Table 2).

In 2007, McCrindle et al. investigated 108 Fontan patients (age 6–18 years) with accelerometers over at least 3 days, and found that physical activity levels were reduced after Fontan procedure [10]. This is discussed in several studies without matched controls from the same area [9, 13, 19], which is important because the place of residence might have an impact on activity levels and exercise capacity. Duppen et al. reported higher $\dot{V}O_{2\mathrm{peak}}$ values measured on a bicycle ergometer in Dutch Fontan patients, who were used to cycling in everyday life. Müller et al. found, that Fontan patients from Munich, Germany, aged 11–18 years, all reached the daily activity recommendations of the United Kingdom Expert Consensus group (MVPA ≥60 min, ≥5d/week) [9]. In 2012, he also showed a normal daily activity pattern in 42 adult patients [19].

Although our Fontan group achieved lower activity levels than the Munich cohort, activity level compared to matched controls was not diminished. Nevertheless, time spent in MVPA in both teenager groups was alarmingly low. These results are in concordance with findings from the KiGGS-study, where the proportion of children (14–17 years) reaching the WHO recommendations was similar [20]. Therefore, physical activity should be promoted in Fontan patients and healthy teenagers.

### CPET and MVPA

With approximately 63% of peak oxygen uptake in contrast to controls, our $\dot{V}O_{2\mathrm{peak}}$ values concur with results from other studies [4, 13, 21, 22].

In line with previous findings [9, 16], Fontan patients differ from controls in that time spent in MVPA correlated significantly with $\dot{V}O_{2\mathrm{peak}}$ values. This could be explained by Hollmann et al., who found that healthy women and men physiologically reach their maximum oxygen uptake between the age of 15–17 years and 18–19 years, respectively. The $\dot{V}O_{2\max}$ values remain stable until the age of 30 years, when exercise capacity starts to decrease without training [23]. In Fontan patients, the cardiopulmonary system and muscles need to be trained daily to maintain physical capacity, because cardiopulmonary capacity diminishes disproportionately from early adulthood on [9]. These findings are supported by our capillary lactate measurements. Especially at lower workloads (0.5 W/kg, 1.0 W/kg), affecting intensity of everyday life activity, patients with higher values of daily MVPA had lower lactate levels at defined workloads and the highest oxygen uptake. At a workload of 1.5 W/kg we also observed this trend, but due to a broad distribution, the correlation was not significant. Since lactate production is associated with training condition and skeletal muscle fatigue [24], daily MVPA has an effect on subjective exhaustion in everyday activities. In this respect, skeletal muscle becomes even more important, and may play a major role in exercise limitations in this patient group [4, 8, 25]. Therefore, we assume that exercise training of leg muscles not only leads to muscle hypertrophy and consequently a higher preload [8, 26], but also results in lower lactate concentrations at similar workloads, as described for healthy individuals [23].

In addition, since light activity does not seem to have similar effects (e.g. no correlation between frequency of light activity and $\dot{V}O_{2\mathrm{peak}}$ or between number of steps and $\dot{V}O_{2\mathrm{peak}}$), parents should be enlightened about the importance of a regular active lifestyle with at least moderate intensities of physical activity in Fontan patients.

Further studies are required to investigate whether regular moderate, high intensity or strength training is best for patients to improve cardiopulmonary capacity and quality of life.

### Heart rate behaviour at rest, exercise and during everyday life

Although resting heart rates are similar in Fontan patients and controls, peak heart rates differ significantly with physical exercise (Table 3). Several studies describe a chronotropic incompetence, which might be caused by extensive atrial surgery, beta-blockers and pacemaker therapy [4, 27]. We also saw significantly higher peak heart rates in controls during CPET. Interestingly, patients on beta-blockers did not demonstrate the lowest peak heart rates during exercise, and there was no difference to those without beta-blocker medication. Pacemakers did not affect peak heart rates in our cohort.

Nevertheless, it should be mentioned that Fontan patients reached peak heart rates at significantly lower workloads. For this reason, peak values only show limited comparability. Accordingly, we compared heart rates at defined workloads and found a similar response for increasing workloads up to 2 W/kg (Fig 4E). This concurs with our findings on daily heart rate behaviour, where we demonstrated that an increase in hourly MVPA resulted in similar increases in heart rates (Fig 4A–4D). In the majority of our investigated patients, there was no difference in heart rate response on increasing exercise compared to controls, and chronotropic incompetence did not seem to be a limiting factor for exercise below workloads of 2 W/kg and exercise tolerance. It is still not clear whether an impaired heart rate response and thereby chronotropic incompetence is relevant above this threshold.

In addition, only control adults showed significantly higher peak heart rates during CPET, whereas Fontan teenagers showed similar or even higher peak heart rates in daily life (Fig 6). This suggests that Fontan patients did not reach their individual maximum physical capacity during CPET, either due to muscular exhaustion or lack of motivation, which also explains lower peak lactate levels during CPET in the Fontan group. Alternatively, some sports (e.g. running) achieve a higher maximum heart rate than cycling [28], and this could be why similar peak heart rates were recorded in both teenager groups (which reported mainly running activities, while tracking) compared to peak heart rates during CPET.

Additional studies are needed to determine whether training Fontan patients can improve exercise performance above workloads of 2 W/kg and thereby lead to a further increase in peak heart rates, or if chronotropic incompetence really is a limiting factor for cardiopulmonary capacity.

The results from our CPET reconfirmed that higher intensities of physical activity are safe for the majority of Fontan patients, since all spiroergometric and cardiopulmonary parameters did not show abnormalities in individual ranges. Fontan patients had significantly lower peak heart rates, but the heart rate responses at defined workloads were equal. The majority of the investigated Fontan patients did not show a chronotropic incompetence until 2 W/kg. Many of them also reached similar or even higher peak heart rates in everyday life as seen during maximum exhaustion in CPET.

## Conclusions

Our study showed that daily physical activity frequencies and intensities are alarmingly low and do not differ between Fontan patients and controls. An active lifestyle should therefore be promoted, since MVPA plays an important role in maintaining and improving exercise capacity from childhood on, especially in Fontan patients. Therefore, higher intensity activities should be implemented regularly in everyday life as part of a multimodal approach, in order to positively influence oxygen uptake and lactate metabolism, muscle function and endurance performance. Excluding children with Fontan circulation from school or team sports is not acceptable. This is in accordance to data from Hedlund et al. and a review by Cordina et al., who recently assumed that endurance training, amongst others, improves physical capacity of these patients and is beneficial [29, 30].

To provide better recommendations, more data from interventional studies are needed to investigate whether sports in moderate or possibly even vigorous activity (e.g. high-intensity interval-training) improve exercise capacity the most.

### Study limitations

A heterogenous group of Fontan patients was chosen to illustrate the broad spectrum of underlying heart failures. Nevertheless, with 18 included patients’ activity data sets, the group size might not represent all Fontan patients.

Although activity tracking with devices such as the Actigraph wGT3x-BT is widespread, the authors needed to define threshold as standardised cut-off points have not yet been agreed. Therefore, widely different classifications of measured activity data exist, increasing the chance of overestimating or underestimating MVPA, which makes comparison of our results with other activity studies difficult. In addition, due to the study situation, probands might show different activity patterns.

With average wear-times of approximately 10 hours per day, the included data did not differ between the groups and were representative. Nonetheless, in order to get a sufficient number of data-sets from activity tracking, we defined a minimal daily wear-time of 330 minutes.
